# Proceedings of the Miss. Valley Association Dental Surgeons

**Published:** 1852-01

**Authors:** 


Proceedings of the Mississippi Valley Association of Dental Surgeons.—Our attention was called, a day or two since, to the omission of the report offered by the minority of the committee in relation to Dr. Allen's improvement. We are not able to find this report among our papers, or we should publish it. The report took ground against the claims of Dr. Allen, and contained a censure for the manner in which it was brought before the profession.

